# Rapid and Near Real-Time Assessments of Population Displacement Using Mobile Phone Data Following Disasters: The 2015 Nepal Earthquake

**DOI:** 10.1371/currents.dis.d073fbece328e4c39087bc086d694b5c

**Published:** 2016-02-24

**Authors:** Robin Wilson, Elisabeth zu Erbach-Schoenberg, Maximilian Albert, Daniel Power, Simon Tudge, Miguel Gonzalez, Sam Guthrie, Heather Chamberlain, Christopher Brooks, Christopher Hughes, Lenka Pitonakova, Caroline Buckee, Xin Lu, Erik Wetter, Andrew Tatem, Linus Bengtsson

**Affiliations:** Flowminder Foundation, Stockholm, Sweden; Geography & Environment, University of Southampton, Southampton, UK; Flowminder Foundation, Stockholm, Sweden; Geography & Environment, University of Southampton, Southampton, UK; Flowminder Foundation, Stockholm, Sweden; Faculty of Engineering & the Environment, University of Southampton, Southampton, UK; Flowminder Foundation, Stockholm, Sweden; Electronics & Computer Science, University of Southampton, Southampton, UK; Flowminder Foundation, Stockholm, Sweden; Electronics & Computer Science, University of Southampton, Southampton, UK; Flowminder Foundation, Stockholm, Sweden; Electronics & Computer Science, University of Southampton, Southampton, UK; Flowminder Foundation, Stockholm, Sweden; Politics & International Relations, University of Southampton, Southampton, UK; Flowminder Foundation, Stockholm, Sweden; Geography & Environment, University of Southampton, Southampton, UK; Flowminder Foundation, Stockholm, Sweden; Geography & Environment, University of Southampton, Southampton, UK; Flowminder Foundation, Stockholm, Sweden; Electronics & Computer Science, University of Southampton, Southampton, UK; Oxford Internet Institute, University of Oxford, Southampton, UK; Flowminder Foundation, Stockholm, Sweden; Electronics & Computer Science, University of Southampton, Southampton, UK; Flowminder Foundation, Stockholm, Sweden; Center for Communicable Disease Dynamics, Harvard T.H. Chan School of Public Health, Boston, USA; Department of Epidemiology, Harvard T.H. Chan School of Public Health, Boston, USA; Flowminder Foundation, Stockholm, Sweden; Department of Public Health Sciences, Karolinska Institute, Stockholm, Sweden; College of Information System and Management, National University of Defense Technology, Changsha, China; Flowminder Foundation, Stockholm, Sweden; Stockholm School of Economics, Stockholm, Sweden; Flowminder Foundation, Stockholm, Sweden; Geography & Environment, University of Southampton, Southampton, UK; Flowminder Foundation, Stockholm, Sweden; Dept. of Public Health Sciences, Karolinska Institute, Sweden

## Abstract

Introduction: Sudden impact disasters often result in the displacement of large numbers of people. These movements can occur prior to events, due to early warning messages, or take place post-event due to damages to shelters and livelihoods as well as a result of long-term reconstruction efforts. Displaced populations are especially vulnerable and often in need of support. However, timely and accurate data on the numbers and destinations of displaced populations are extremely challenging to collect across temporal and spatial scales, especially in the aftermath of disasters. Mobile phone call detail records were shown to be a valid data source for estimates of population movements after the 2010 Haiti earthquake, but their potential to provide near real-time ongoing measurements of population displacements immediately after a natural disaster has not been demonstrated.

Methods: A computational architecture and analytical capacity were rapidly deployed within nine days of the Nepal earthquake of 25th April 2015, to provide spatiotemporally detailed estimates of population displacements from call detail records based on movements of 12 million de-identified mobile phones users.

Results: Analysis shows the evolution of population mobility patterns after the earthquake and the patterns of return to affected areas, at a high level of detail. Particularly notable is the movement of an estimated 390,000 people above normal from the Kathmandu valley after the earthquake, with most people moving to surrounding areas and the highly-populated areas in the central southern area of Nepal.

Discussion: This analysis provides an unprecedented level of information about human movement after a natural disaster, provided within a very short timeframe after the earthquake occurred. The patterns revealed using this method are almost impossible to find through other methods, and are of great interest to humanitarian agencies.

## Introduction

On average, twenty-six million people per year have been displaced by natural disasters between 2008 and 2014 1. Displaced people are in many disaster settings the most vulnerable group and frequently most in need of assistance 2 . However, traditional methods of quantifying large-scale population movements after disasters are slow and unreliable 3 ^,^ 4 ^,^ 5. This is complicated by the fact that displaced people frequently move into urban informal settlements or seek shelter with host families, where they are effectively lost to follow-up from humanitarian agencies 6.

De-identified mobile operator data have the potential to shed light on population movement and displacement patterns. Operationally they were used in the aftermath of the Haiti 2010 earthquake to quantify population mobility and displacement patterns, in the Haiti 2010 cholera outbreak outputs to inform the early response activities^7^ and during the 2014 Ebola outbreak to model pre-outbreak mobility patterns in affected countries^8^. In Haiti, the relative geographic distribution of mobile phone movements correlated closely with data from a large-scale retrospective household survey, performed seven months after the earthquake^7^. However, the first analyses for responders to the Haiti earthquake were distributed four months after the earthquake, too late to support the early humanitarian response^6^. Similar analyses after a natural disaster, based on mobile operator data, were performed after the 2011 earthquake in Christchurch, New Zealand but were first released 12 months after the earthquake^9^.

De-identified mobile phone call detail records (CDRs) contain the time and associated cell tower of text messages and calls and thus can be used to study human behaviour and mobility patterns^8^^,^ 10 ^,^ 11. CDRs are electronic records kept by telecommunication providers that contain individual level data on mobile phone usage. The data typically consists of a record for each ‘event’ - such as making or receiving a call, or sending or receiving a text message - with attributes, such as a timestamp, duration, and - crucially - the cell tower that the user is connected to at the time^12^. CDRs thus provide rich information for measuring the collective mobility patterns of populations over time^11^. Although there are inherent biases within the data due to differences in phone ownership across geographical and socioeconomic groups, evidence so far suggest that CDRs currently provide by far the best - and most current - data available to describe population movement patterns in low and middle-income countries^7^^,^ 8 ^,^ 13. CDR-based mobility data, therefore, have been used across a variety of research areas; for example, prediction and modelling of the spread of infectious diseases^14^^,^ 15 ^,^ 16 ^,^ 17 ^,^ 18 ^,^ 19, traffic monitoring^20^ and the analysis of commuting patterns^21^.

Here, we report on ongoing work following the 2015 Nepal earthquake, where estimates of national level population movements based on de-identified data from 12 million mobile phones were released to all parties within nine days of the earthquake. The work was made possible by the establishment of a collaboration with the operator before the earthquake. Details of the analysis technical framework, population movement patterns after the earthquake and areas for future research will be discussed.

## The 2015 Gorkha earthquake

The Gorkha earthquake, named from the district in which it originated, struck on the 25th April 2015 at 11.56 am local time. The quake had an intensity of 7.8Mw with an epicentre at 28°14'24''N, 84°45'0''E^22^. It triggered massive avalanches on Mount Everest and in the Langtang Valley. Aftershocks occurred at 15-20 minute intervals during the following days^23^. A 6.9ML secondary earthquake (epicentre 27°50'24''N, 86°2'60''E), occurred in the Dolakha District a day later, at 12:54 pm local time. A series of two more major earthquakes and four aftershocks hit Dolakha District once again on 12th May 2015, with the two earthquakes registering 6.8ML and 6.2ML^22^. By the 23rd June 2015, the Nepali Government stated that Dolakha District had the highest number of casualties outside the Kathmandu Valley^24^.

As of 1st June 2015, the Nepali Government estimated the earthquake to have caused 8,673 deaths and 22,309 injuries^24^^,^ 25. The UN Resident Coordinator's Office in Nepal estimated that 2.8m people were in need of immediate assistance^26^. Significant landscape shifts, a severe degradation of Nepal’s transport network, numerous landslides and avalanches along the Kathmandu Valley resulted in large parts of the country becoming isolated.

The destruction of villages and towns in the most severely-affected regions further contributed to population displacement, compounded by severe damages to transport infrastructure^27^. The Nepali government estimates 2,600 government buildings were completely destroyed in the earthquakes, with some 3,700 more partially destroyed^24^.

## Methods

**Mobile phone use in Nepal**

Nepal has 26 million mobile phone subscriptions^28^ and a population of 27 million people^29^. The two largest operators, Ncell and Nepal Telecom, have 12.9 and 12.2 million subscribers respectively^30^. Mobile phone penetration is increasing rapidly: in 2011, 75% percent of households (92% in urban areas, 72% in rural areas) reported having at least one mobile phone^31^ .

**Setup**

The Flowminder Foundation develops methods for estimating mobility patterns and population displacement. It has established collaborations with mobile phone operators in countries where analyses of de-identified mobile operator data can support preparedness and response to humanitarian disasters. An agreement between Flowminder and Ncell in Nepal was signed 6 months before the earthquake. The first technical planning meeting between the parties took place in Kathmandu one week prior to the earthquake. Although the technical set-up had not been completed at the time of the earthquake, Ncell were able to provide access to de-identified data for Flowminder analyses within six days of the earthquake.

CDR analysis was undertaken in compliance with the GSMA privacy guidelines developed in the context of the Ebola outbreak^32^. These guidelines state that analyses should be performed on de-identified data and that individual-level data should not be transferred from the mobile phone operator’s servers. Therefore, we collaborated with Ncell to set up a high-specification Linux analysis server (with 128GB of RAM and over 20TB of disk space) within their data centre. All analyses were performed by connecting to this server remotely, analysing the data, and then only transferring aggregated data outside the operator.

The analysis framework was developed in the Python programming language, and consisted of a series of preprocessing steps automatically run for each new day’s CDR data, followed by separate analyses designed to investigate particular aspects of mobility after the earthquake. Analysis was performed using custom-written code, which used the standard scientific Python stack^33^^,^ 34 ^,^ 35.

**Preprocessing**

Each day’s CDRs were provided by 1am on the following day and historical data back to January 2015 were also provided. The preprocessing steps were designed to reduce this raw CDR data (which was provided as approximately 12GB of CSV files per day) to a more manageable size, which consequently lowers the time and memory usage of the subsequent analyses significantly. The preprocessing involved:

1. Shrinking the raw data by removing unused information, and assigning each separate tower location a *location ID*, thus reducing the data to around 2.5GB per day.
2. Calculating a ‘daily location’ for each user (where a user is taken to be a unique phone number, i.e. a SIM card). This was designed to be a single tower location which represents the location of the user for that day. As the aim was to investigate displacement, the overnight location of a user determined their daily location. Investigations were made into how best to estimate this location from the location of all calls a user made that day, including subsetting by time, or spatially grouping calls. However, in Nepal, the most appropriate definition was judged to be simply the location of the last call the user made that day.
3. Assigning daily locations on administrative boundary level based on the tower level location. In this work administrative boundaries at level 3 (District) and level 4 (Village Development Committee, or VDC) were used, digitised from Nepali governmental maps by the UN Organisation for the Coordination of Humanitarian Affairs^36^^,^ 37 ^,^ 38. Each user was assigned a daily location at District or VDC level, based on the administrative area that the daily location (at tower level) was situated in.

**Estimating population flows**

Conceptually, flows can be estimated by simply recording a location for each user at two different times, and then counting the number of users who moved from one location to another. This produces a *transition matrix* containing the flow of users between each possible pair of locations^7^^,^ 11. The origin and arrival locations can be defined at the tower-level or aggregated by administrative boundary level (District or VDC) as described above. To describe displacements, the ideal transition matrix includes moves from people’s homes to a new temporary or permanent location and ignores short term movements such as a daily commute. Therefore, choosing the right spatial and temporal scale to capture movements indicating moves or displacements is crucial. To reduce the influence of noise introduced by short term trips or commuting patterns a ‘home location’ was calculated for each individual by calculating the modal daily location over a certain period. These home locations were then used to calculate transition matrices describing the countrywide mobility between two points in time.

In Nepal, mobility is fairly high and thus large flows of people are observed between areas of Nepal under normal conditions. To account for this high baseline mobility, flows following the earthquake (termed *post-earthquake flows*) were normalised using pre-earthquake mobility estimates (*normal flows*). Normal flows were calculated as the changes in location from a *benchmark period* (consisting of historical data from the 1st January until 7th April 2015) to a *comparison period* just before the earthquake (20th-24th April, chosen to avoid the large population flows that may be experienced around the Nepali New Year festival). Post-earthquake flows were calculated between the benchmark period and the *focal period*, which is the period of interest, the most recent week of data (Figure 1)***.***

The difference between post-earthquake flows and normal flows provides a measure of anomalous flows or 'flows above/below normal'. An important added value of this approach is that most of the noise in the estimates of home locations due to high levels of daily mobility under normal conditions is cancelled out. This set-up allows examination of how the earthquake has affected mobility without erroneously attributing high but nevertheless normal flows, generated by overall high mobility, to the earthquake. Flows are only calculated for users who make calls in all three of these periods, thus excluding SIM cards lost during the earthquake, and incoming relief workers.

The flows calculation produced a *transition matrix* giving the anomalous flow (number of users, above and below normal) moving between each pair of locations. This can be summarised to produce two metrics for each region: the total above-normal inflows (the sum of all of the flows from any region into that region), and the total above-normal outflows (the sum of all the flows from that region into any other region).

**Diagram showing the periods used for calculating anomalous flows:**
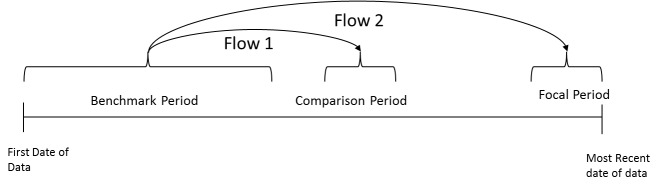

**Scaling population flows**

Humanitarian agencies require information about total population flows, rather than the subset represented by subscribers. Assuming SIM card movements to be representative of population movements, absolute flows were estimated by scaling SIM cards counts based on local Ncell user penetration rates. The number of active SIM cards in an area, for example an administrative unit, was calculated from CDRs. Pre-earthquake census-based population data is however not available per tower area or for small administrative areas. Data from WorldPop, which provides gridded population estimates per 100x100 meter grid square for the entire country, was used to estimate finer level population counts^39^ (http://www.worldpop.org.uk/).

For each District (administrative level 3), WorldPop population counts for 2015, adjusted to match UN estimates at the national level, were summed to produce administrative-level population estimates. Scaling factors, used to estimate absolute population flows were then calculated at District level. It was assumed that the ratio of the flow of SIM cards \begin{equation*}\vec{xy}\end{equation*} to the combined number of SIM cards (\begin{equation*}x + y\end{equation*} ), is representative of the ratio of the actual population flow \begin{equation*}\vec{XY}\end{equation*} to the combined population (\begin{equation*}X + Y\end{equation*} ). This can be formalised as:

Mathematical formalisation of scaling assumption \begin{equation*}\frac{\vec{XY}}{X+Y} = \frac{\vec{xy}}{x+y}\end{equation*}

resulting in the following scaling factor used in the analyses:

Scaling factor derivation \begin{equation*}\vec{XY} = \frac{\vec{xy}(X+Y)}{x+y}\end{equation*}

This was validated for the context of Nepal by comparing the scaled estimates of total inflows (including static users) for each area during the pre-earthquake baseline period with the census population data for that area, which showed a close match.

To understand how flows were changing in the weeks following the earthquake, anomalous flows were calculated for each week of interest, with the focal period gradually moving forward in time.

**Analyses of returning residents**

An additional question of importance to relief work is identifying regions to which people have not returned, as this may indicate regions in which recovery has not yet reached sufficient levels. One way to assess this is to identify users who have left their home location due to the earthquake, and continue to reside in another location.

This can be done by determining the home location of users over a long benchmark period prior to the earthquake (1st January until 7th April 2015 in this case), using the same method as for the flows calculation above. A user was counted as displaced if they had spent at least seven consecutive days away from their pre-earthquake home location in a two week period after the earthquake. Iterating through the remaining data the percentage of displaced users who remained away (i.e. at a location different to the pre-earthquake home location) was calculated.

Plotting the percentage of users who had not returned over time gives an indication of the rate at which users are returning to a given location. As this is a percentage it does not suffer from the uncertainties that scaling may introduce in the analyses above. Over time, a portion of users disappear from the data set. This may be because they have left Nepal or their SIM card has become inactive. We assumed that the same percentage of missing users remained away from home as those who were present in the data set.

Using this data, trends in the ‘return rate’ for each region can be derived as well as snapshots of the most recent data. In the latter case regions are coloured based on the mean and standard deviation of the dataset, marking regions with a high (\begin{equation*}x > (\mu + \sigma)\end{equation*}), medium (\begin{equation*}(\mu - \sigma) < x < (\mu + \sigma)\end{equation*}) and low (\begin{equation*}x < (\mu - \sigma)\end{equation*}) proportion of people still away from home.

## Results

The first preliminary results were available nine days after the earthquake, with the first full report released thirteen days after the earthquake. In this time the server was set up, the preprocessing and analysis code designed and written, all data processed, and the outputs checked for accuracy.

The first few weeks after the earthquake saw large flows out of the Kathmandu Valley area to surrounding areas (particularly to Nuwakot and Kavrepalanchok) as well as to the highly-populated areas in the central southern region of Nepal (Figure 4). Overall, an estimated 390,000 people above normal levels had left the valley. Flows from Kathmandu to the areas in the North were still higher than normal, but significantly lower than those to the South - likely due in part to the higher level of earthquake damage in the northern regions.

Looking at the flows into each region over time, from just after the earthquake until mid-July (Figure 5) shows sharply decreased flows into Kathmandu Valley immediately after the earthquake. This reduction gradually normalised. By early June the flows were very close to normal conditions, and by late June the estimated number of people in Kathmandu Valley had increased to above the pre-earthquake level (nearly 50,000 additional people had come into the Kathmandu Valley compared to pre-earthquake levels). This increase may be influenced by normal seasonal movements but may also be caused by the ongoing reconstruction work in the Kathmandu Valley.

The other regions can be categorised into three groups: those which experienced little changes in flows due to the earthquake (Okhaldhunga and Rasuwa, with inflows around 5,000 people above normal), those which experienced very large inflows immediately after the earthquake (Nuwakot and Gorkha, with inflows around 30,000 people above normal) and the remaining regions, which experienced a moderate increase in flows (with inflows around 10,000-25,000 people above normal) immediately after the earthquake. Flows in all regions (excluding the Kathmandu Valley) seem to have stabilised since late June.

**Anomalous flows from the Kathmandu valley, comparing the 10th-14th May with the 20th-24th April:**
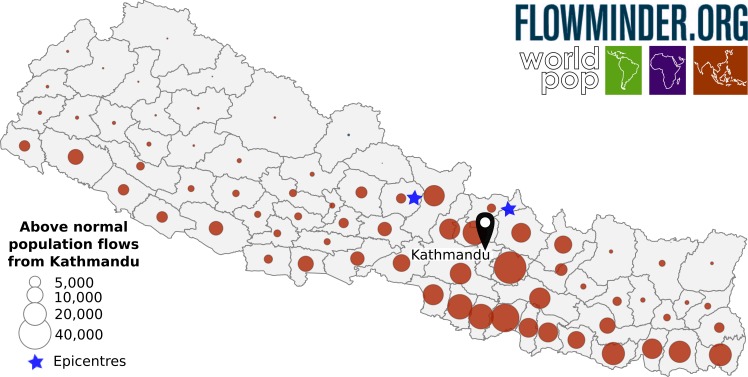

**Anomalous inflows (above normal) for the ten focus Districts (note the two y axes to deal with the significantly higher values for Kathmandu):**
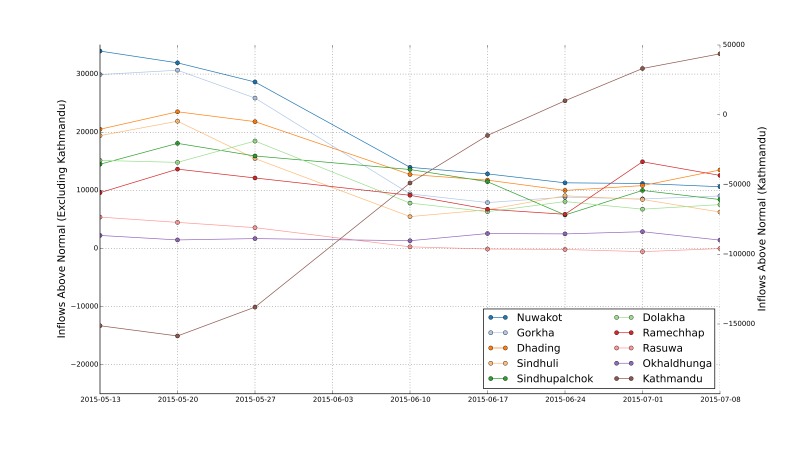

The trends in return rates over time (Figure 6) show a significant decline over time for all regions, with almost 50% of the people displaced still away in early May (not shown in graph, for clarity) and a maximum of 15% still away in late July. Some regions are doing notably better (Dhading and Gorkha) and some notably worse (Bhaktapur and Kathmandu) than average. Some regions have recovered more quickly than others, and some regions have relatively sudden changes (for example, Dolakha in early July), which may coincide with recovery work in these regions.

**Percentage of people who left their home district who remain away, over time from early May after the earthquake until the end of July:**
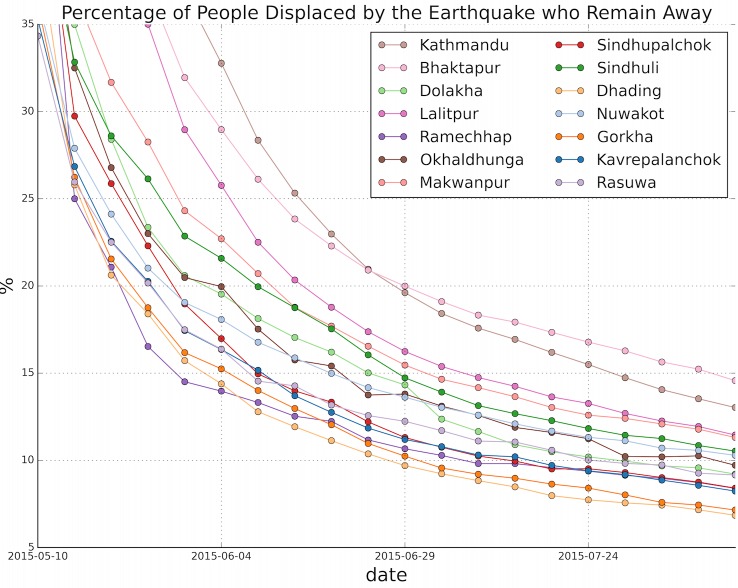

Examining this spatially, at VDC-level (Figure 7), shows which regions are performing particularly well, or particularly poorly. Clusters of regions with low return rates can be seen in the outskirts of Kathmandu (potentially suggesting a focus of recovery on the city centre), regions to the south-west of Kathmandu and a number of the regions in the mountainous northern regions. While mobile coverage of the population was good, many areas are mountainous and sparsely populated. Areas with no coverage or insufficient data are marked in grey.

**Percentage of people displaced by the earthquake who remain away as of the 19th August, shown spatially for the focus Districts, at VDC-level.**
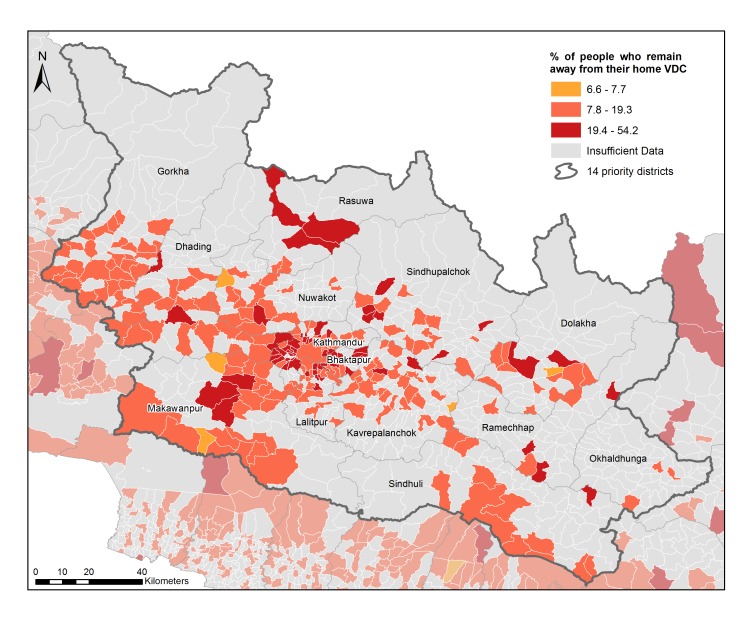

## Discussion

We showed that an estimated 390,000 people above normal left the Kathmandu Valley soon after the earthquake. Many of these moved to the surrounding areas, and the highly-populated areas in the central southern area of Nepal. People who left their home area after the earthquake have gradually returned, with the return rate varying between regions. By late July, all Districts had less than 15% of people still away from their original home location, with some as low as 5%.

The analyses presented above have provided an unprecedented level of information about human displacement after the Nepal earthquake. This data has never been available before in such a short time after a natural disaster has occurred, which was made possible by having a data access agreement in place as well as the operator providing rapid access to the data very soon after the earthquake. The analyses reveal national level population mobility patterns and return rates which are extremely difficult or impossible to acquire using other methods. These are of great relevance to humanitarian agencies, as mobility patterns can help identify where aid should be directed, and low return rates can identify areas where recovery and reconstruction work may not be progressing well.

Reports containing these results, along with interpretation, were distributed to relevant humanitarian agencies working on the ground in Nepal, and are available online at the WorldPop website (www.worldpop.org.uk/nepal/). Key results of analyses were included in reports by the UN Resident Coordinator^40^.

The analyses have several limitations, some of which were due to the speed with which the analyses needed to be delivered and several of which are possible to address in future studies. Phone ownership is skewed towards certain population groups: potential biases include higher ownership of phones among males than females as well as among higher income groups and certain age groups^41^^,^ 42 ^,^ 43. Similarly, phone usage within households can vary, and phones can be shared between multiple members of a household. While the scaling method described above is likely to account for some of these biases, estimates could be further improved by incorporating information on phone ownership from surveys in cases where those are available. It is also currently unknown to what extent family members without phones moved together with family members with phones.

Furthermore, analyses estimate the number of people moving after the earthquake but not the reasons for doing so. While estimation of population flows above and below normal levels aims to address this issue, a higher precision in displacement estimates would have been possible if analyses had been combined with population surveys. Such surveys are now being planned as well as comparison between CDR-derived displacement estimates and survey-based data collected by third parties.

CDRs only provide location updates for individuals when a call is made. In this dataset calling frequencies were relatively low, with around 50% of people calling at least every other day. Detailed movements among users with infrequently updated locations are therefore missing from the data 44. However, the effects on the analyses are reduced when home locations are calculated using the modal value of multiple consecutive daily locations.

A small proportion of SIM cards become inactive over time. New SIMs tend to enter the dataset at roughly the same rate but there is no way of linking the old and new SIMs to a particular user. Most analyses can be corrected to account for this effect, but this may cause bias if the data is analysed over very long time frames.

In these analyses we assumed that towers belong to the administrative area in which they are placed, as tower functionality and replacements took place repeatedly during the period. Coverage areas do however often extend over more than one administrative area, which may have contributed to bias. This can potentially be improved by dynamically accounting for changing tower coverage areas in the analyses. For many VDC areas little or no data is available. The principle reason for this is that the mobile network did not have sufficient coverage in these regions to make statistically significant inferences. However, while these areas geographically make up a large proportion of the area of Nepal, they are mostly concentrated in the sparsely populated mountainous areas with small population densities. We were not able to take into account seasonal drivers of population movements. Ideally, one or several years of historical data would be used to adjust for seasonality in these mobility patterns.

Avouac et al. (2015)^45^ concludes that the Ghorka earthquake in Nepal has not released all of the stress from the tectonic plates in the region. Another earthquake is therefore likely to occur within the next few years, and Nepal routinely experiences large floods and landslides. Our ongoing work is focusing on further automating the analyses processes and improving estimation methods to allow us to rapidly provide high-quality estimates of population displacement.

## Conclusions

The value of CDRs when integrated with more traditional data sources within modelling frameworks has been shown across multiple disease, development and disaster application examples. The field of CDR analytics in the humanitarian space is therefore moving beyond pilot studies and towards a more mature and operationally valuable platform. Here we have shown how this can be achieved in a disaster response situation through the partnership of scientists, mobile network operators and response agencies. The work described is not a single study, but the initiation of an ongoing dynamic and near-real time monitoring system, providing data support to a country with high levels of poverty, and populations that are highly vulnerable to the effects of natural disasters and disease outbreaks.

## Competing Interests

The authors have declared that no competing interests exist.

## References

[1] Yonetani, M., Lavell, C., Bower, E., Meneghetti, L. And O’Conner, K (2015) Global Estimates, People displaced by Disasters, International Displacement Monitoring Centre. Available at: http://www.internal-displacement.org/assets/library/Media/201507-globalEstimates-2015/20150713-global-estimates-2015-en-v1.pdf, Accessed 30.10.2015.

[2] King, D (2002) You’re on your own: Community Vulnerability and the Need for Awareness and Education for Predictable Natural Disasters, Journal of Contingencies and Crisis Management, 8(4), 223-228.

[3] Schimmer, R (2010) Tracking the genocide in Darfur: population displacement as recorded by remote sensing, New Haven (Connecticut): Genocide Studies Program. Yale University. Available at: http://www.yale.edu/gsp/gis-files/darfur Accessed 9 December 2010, Accessed 07.12.2015.

[4] NRCC (1998) The demography of forced migration: summary of a workshop, National Research Council Committee on Population. Washington (D.C.): National Academy Press.

[5] Brown, V., Jacquier, G., Coulombier, D., Balandine, S. And Belanger, F (2001) Rapid assessment of population size by area sampling in disaster situations, Disasters 25, 164–171. 10.1111/1467-7717.00168 11434235

[6] Lu, X., Bengtsson, L. And Holme, P (2010) Predictability of population displacement after the 2010 Haiti earthquake, Proceedings of the National Academic of Sciences of the United States of America, 109(29), 11576-11581. 10.1073/pnas.1203882109 PMC3406871 22711804

[7] Bengtsson, L., Lu, X., Thorson, A., Garfield, R. And Screeb, J (2011) Improved response to disasters and outbreaks by tracking population movements with mobile phone network data: a post-earthquake geospatial study in Haiti, PLoS Med, 8(8). 10.1371/journal.pmed.1001083 PMC3168873 21918643

[8] Wesolowski, A., Stresman, G., Eagle, N., Stevenson, J., Owaga, C., Marube, E., Bousema, T., Drakeley, C., Cox, J. And Buckee, C (2014) Quantifying travel behavior for infectious disease research: a comparison of data from surveys and mobile phones, Scientific Reports, (4), 5678. 10.1038/srep05678 PMC4894426 25022440

[9] Statistics New Zealand (2012) Using cellphone data to measure population movements: Experimental analysing following the 22 February 2011 Christchurch earthquake. Available at: http://www.stats.govt.nz/tools_and_services/earthquake-info-portal/using-cellphone-data-report.aspx, Accessed 4.11.2015.

[10] Oliver, N., Matic, A. And Frias-Martinez, E (2015) Mobile Network data for public health: opportunities and challenges, Public Health. Available at: http://dx.doi.org/10.3389/fpubh.2015.00189, Accessed 30.11.2015. 10.3389/fpubh.2015.00189 PMC4528087 26301211

[11] Gonzalez, M., Hidalgo, C. And Albert-Laszlo, B (2008) Understanding individual human mobility patterns, Nature, 453, 779-782. 10.1038/nature06958 18528393

[12] Horak, R (2007) Telecommunications and data communications handbook. Wiley: New York.

[13] Wesolowski, A., Eagle, N., Abdisalan, M.N., Snow, R.W. And Buckee, C (2013) The impact of biases in mobile phone ownership on estimates of human mobility, Royal Society Publishing, 10(81). 10.1098/rsif.2012.0986 PMC3627108 23389897

[14] Frias-Martinez, V., Rubio, A. And Frias-Martinez, E (2012) Measuring the impact of epidemic alerts on human mobility, Pervasive Urban Applications–PURBA. Available at: http://dl.acm.org/citation.cfm?id=2497350, Accessed 8.12.2015.

[15] Tatem, A.J., Qiu, Y., Smith, D.L., AS,A. And Moonen, B (2009) The use of mobile phone data for the estimation of the travel patterns and important Plasmodium falciparum rates among Zanzibar residents, Malaria Journal, 8 (287). 10.1186/1475-2875-8-287 PMC2800118 20003266

[16] Wesolowski, A., Eagle, N., Tatem, A.J., Smith, D.L., Noor, A., Snow, R. And Buckee, C (2012) Quantifying the impact of Human Mobility on Malaria, Science, 338(6104), 267-270. 10.1126/science.1223467 PMC3675794 23066082

[17] Tatem A.J., et al (2014) Integrating rapid risk mapping and mobile phone call record data for strategic malaria elimination planning, Malaria Journal, 13 (52). 10.1186/1475-2875-13-52 PMC3927223 24512144

[18] Bengtsson, L., Gaudart, J., Lu, X., Moore, S., Wetter, E., Sallah, K., Rebaudet, S. And Piarroux, R (2015) Using Mobile Phone Data to Predict the Spatial Spread of Cholera, Scientific Reports, 5 (8923). 10.1038/srep08923 PMC4352843 25747871

[19] Wesolowski, A., Metcalf, C.J., Eagle, N., Kombich, J., Grenfell, B., Bjornstad, O., Lessler, J., Tatem, A.J. And Buckee, C (2014) Quantifying seasonal population fluxes driving rubella transmission dynamics using mobile phone data, PNAS, 112 (35). 10.1073/pnas.1423542112 PMC4568255 26283349

[20] Naboulsi, D., Stanica, R. And Fiore, M (2014) Classifying Call Profiles in Large-scale Mobile Traffic Datasets, IEEE Conference on Computer Communications. Available at: http://ieeexplore.ieee.org/stamp/stamp.jsp?arnumber=6848119, Accessed 8.12.2015.

[21] Frias-Martinez, V., Soguero, C. And Frias-Martinez, E (2012) Estimation of Urban Commuting Patterns Using Cellphone Network Data. In: Proceedings of the ACM SIGKDD International Workshop on Urban Computing, 9–16.

[22] Hayes, G.P., et al (2015) Rapid Characterization of the 2015 Mw 7.8 Gorkha, Nepal, Earthquake Sequence and Its Seismotectonic Context, Seismological Research Letters, 86(6), 1557-1567.

[23] National Seismological Centre (NSC), Government of Nepal: Ministry of Industry. Available at: http://www.seismonepal.gov.np/index.php?action=earthquakes&show=recent&page=5, Accessed 8.12.2015.

[24] Nepal Disaster Risk Reduction Portal (2015) Nepal Earthquake Causalities List, Government of Nepal: Ministry of Science, Technology and Environment. Available at: https://docs.google.com/spreadsheets/u/1/d/1Q3QSx1_p78T4_qo_JVj5vEQtzuCFWP2AfxYnVBSNzLQ/pubhtml, Accessed 8.12.2015.

[25] Government of Nepal (2015) Nepal: Causalities and damages, HDX. Available at: https://ditaanggraeni.cartodb.com/viz/041aa150-f02d-11e4-b4d8-0e018d66dc29/public_map, Accessed 8.12.15.

[26] UNRCO (2015) Nepal Earthquake, HDX. Available at: https://data.hdx.rwlabs.org/group/nepal-earthquake, Accessed 8.12.2015.

[27] Shrestha, S (2015) Langtang is gone, Nepali Times. Available at: http://nepalitimes.com/article/nation/langtang-destroyed-in-earthquake,2205, Accessed 9.12.2015.

[28] Nepal Telecommunications Authority (2015) Management Information Service Report, MIS Reports, 74(122).

[29] United Nations (2015) 2015 Revision of World Population Prospects, Department of Economic and Social Affairs. Available at: http://esa.un.org/unpd/wpp/, Accessed 9.12.2015.

[30] Nepal Telecommunications Authority (2015), NTA-MIS-94 Report, available at http://www.nta.gov.np/en/2012-06-01-11-33-01/mis-archives/mis-reports/nta-mis-94/download

[31] Demographic and Health Survey (2011) Nepal Demographic and Health Survey 2011, Ministry of Health and Population. Available at: http://dhsprogram.com/pubs/pdf/FR257/FR257%5B13April2012%5D.pdf, Accessed 9.12.2015.

[32] GSMA (2014) GSMA guidelines on the protection of privacy in the use of mobile phone data for responding to the Ebola outbreak. Available at: http://www.gsma.com/mobilefordevelopment/wp-content/uploads/2014/11/GSMA-Guidelines-on-protecting-privacy-in-the-use-of-mobile-phone-data-for-responding-to-the-Ebola-outbreak-_October-2014.pdf, Accessed 9.12.2015.

[33] Van Der Walt, S., Colbert, C. And Varoquaux, G (2011) The NumPy array: a structure for efficient numerical computation, Computing in Science and Engineering, 12(12), 22-30.

[34] McKinney, W (2012) Python for data analysis: Data wrangling with Pandas, NumPy, and IPython. O’Reilly Media: California.

[35] Pérez, F. And Granger, B (2007) IPython: a system for interactive scientific computing, Computing in Science and Engineering 9(3), 21-29.

[36] OCHA (2015) Nepal admin level 3 administrative boundaries, HDX. Available at: https://data.hdx.rwlabs.org/dataset/nepal-admin-level-3-administrative-boundaries-cod, Accessed 9.12.2015.

[37] OCHA (2015) Nepal admin level 4 administrative boundaries, HDX. Available at: https://data.hdx.rwlabs.org/dataset/nepal-admin-level-4-administrative-boundaries-cod, Accessed 9.12.2015.

[38] OCHA (2015) Nepal admin level 5 administrative boundaries, HDX. Available at: https://data.hdx.rwlabs.org/dataset/nepal-admin-level-5-administrative-boundaries-cod, Accessed 9.12.2015.

[39] Stevens, F. R., Gaughan, A. E., Linard, C. and Tatem, A. J. (2015), Disaggregating Census Data for Population Mapping Using Random Forests with Remotely-Sensed and Ancillary Data, PLoS One, 10.2: e010704 10.1371/journal.pone.0107042 PMC4331277 25689585

[40] Nepal Earthquake Assessment Unit (2015) Landslides and Displacement Situation Update. Available at: https://www.humanitarianresponse.info/en/system/files/documents/files/150827_landslide_and_displacement_update.pdf, Accessed 9.12.2015.

[41] Wesolowski, A., Eagle, N., Noor, A.M., Snow, R.W. And Buckee, C (2013) The impact of biases in mobile phone ownership on estimates of human mobility, Journal of the Royal Society. Available at: http://rsif.royalsocietypublishing.org/content/royinterface/10/81/20120986.full.pdf, Accessed 9.12.2015. 10.1098/rsif.2012.0986 PMC3627108 23389897

[42] Schlapfer, M., Bettencourt, L.M., Grauwin, S., Raschke, M., Claxton, R., Smoereda, Z., West, G. And Ratti, C (2015) The scaling of human interactions with city size, Journal of the Royal Society. Available at: http://rsif.royalsocietypublishing.org/content/royinterface/11/98/20130789.full.pdf, Accessed 9.12.2015. 10.1098/rsif.2013.0789 PMC4233681 24990287

[43] Deville, P., Linard, C., Martin, S., Gilbert, M., Stevens, F., Gaughan, A., Blondel, V., Tatem, A.J (2014) Dynamic population mapping using mobile phone data, PNAS. Available at: http://www.pnas.org/content/111/45/15888.abstract?tab=metrics, Accessed 9.12.2015. 10.1073/pnas.1408439111 PMC4234567 25349388

[44] Pinelli, F., Lorenzo, G. And Calabrese, F (2015) Comparing urban sensing applications using event and network-driven mobile phone location data, 16th IEEE International Conference on Mobile Data Management, 219-226.

[45] Avouac, J., Meng, Lingsen., Wei, S., Wang, T. And Ampuero, J (2015) Lower edge of locked Main Himalayan Thrust unzipped by the 2015 Gorkha earthquake, Nature Geoscience, 8, 701-711.

